# Evaluation of the antibacterial effect of Epigallocatechin gallate on the major pathogens of canine periodontal disease and therapeutic effects on periodontal disease mice

**DOI:** 10.3389/fmicb.2023.1329772

**Published:** 2024-01-05

**Authors:** Peijia Song, Yibing Hao, Degui Lin, Yipeng Jin, Jiahao Lin

**Affiliations:** ^1^Country National Key Laboratory of Veterinary Public Health and Safety, College of Veterinary Medicine, China Agricultural University, Beijing, China; ^2^China Veterinary Medicine Innovation Center, China Agricultural University, Beijing, China; ^3^China Agricultural University Veterinary Teaching Hospital, Beijing, China

**Keywords:** canine PD, anaerobic bacteria, antibacterial treatment, EGCG, alveolar bone loss

## Abstract

**Background:**

Periodontal disease (PD) is a prevalent oral affliction in canines, with limited therapeutic options available. The potential transmission of oral bacteria from canines to humans through inter-species contact poses a risk of zoonotic infection. Epigallocatechin gallate (EGCG), the principal catechin in green tea polyphenols, exhibits antibacterial properties effective against human PD. Given the clinical parallels between canine and human PD, this study explores the feasibility of employing EGCG as a therapeutic agent for canine PD.

**Methods and results:**

Initially, a survey and statistical analysis of bacterial infection data related to canine PD in China were conducted. Subsequently, the primary pathogenic bacteria of canine PD were isolated and cultivated, and the *in vitro* antibacterial efficacy of EGCG was assessed. Furthermore, verify the therapeutic effect of EGCG on a mouse PD model *in vivo*. The high-throughput 16S rRNA gene sequencing identified *Porphyromonas, Fusobacterium, Treponema, Moraxella,* and *Capnocytophaga* as the genera that distinguishing PD from healthy canines’ gingival crevicular fluid (GCF) samples in China. The anaerobic culture and drug susceptibility testing isolated a total of 92 clinical strains, representing 22 species, from 72 canine GCF samples, including *Porphyromonas gulae*, *Prevotella intermedia*, *Porphyromonas macacae*, etc. The minimum inhibitory concentration (MIC) ranging of EGCG was from 0.019 to 1.25 mg/mL. Following a 7 days oral mucosal administration of medium-dose EGCG (0.625 mg/mL), the abundance of periodontal microorganisms in PD mice significantly decreased. This intervention ameliorated alveolar bone loss, reducing the average cementoenamel junction to the alveolar bone crest (CEJ-ABC) distance from 0.306 mm ± 0.050 mm to 0.161 mm ± 0.026 mm. Additionally, EGCG (0.3125 mg/mL) markedly down-regulated the expression of inflammatory factor IL-6 in the serum of PD mice.

**Conclusion:**

Our research demonstrates the significant antibacterial effects of EGCG against the prevalent bacterium *P. gulae* in canine PD. Moreover, EGCG exhibits anti-inflammatory properties and proves effective in addressing bone loss in a PD mouse model. These findings collectively suggest the therapeutic potential of EGCG in the treatment of canine PD. The outcomes of this study contribute valuable data, laying the foundation for further exploration and screening of alternative antibiotic drugs to advance the management of canine PD.

## Graphical abstract

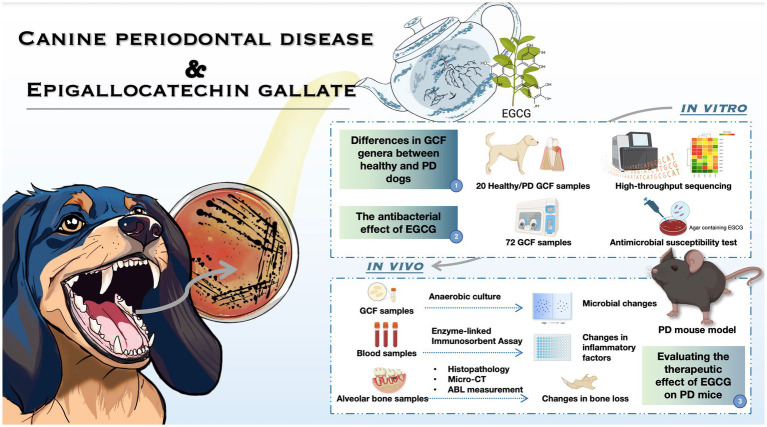

## 1 Introduction

Periodontal disease (PD) is a prevalent, age-related oral disease in canines caused by infectious, inflammatory processes (Kortegaard et al., 2008). While gingivitis is a treatable condition marked by red and inflamed gums, PD involves inflammation of the tissues supporting the tooth, leading to attachment loss through the destruction of the periodontal ligament, cementum, and alveolar bone. In canines, a notable tendency to neglect daily oral hygiene practices contributes to a substantial incidence of PD, affecting approximately 80% of adult canines (Lobprise, 2018; Wallis et al., 2021). This disease can persist, recur, cause discomfort, impede normal canine activities, and diminish overall well-being, with potential correlations to systemic health issues in canines, highlighting the need for effective management.

Comparing human PD with canine PD offers valuable insights due to similarities in clinical presentations. Canine PD, especially its natural-onset form, serves as a relevant animal model for studying human PD (Mangione et al., 2022; Choi et al., 2023). Despite these parallels, the pathogens differ, with *Porphyromonas gingivalis* being the primary pathogen in human PD and *Porphyromonas gulae* in canine PD. But there are similarities in the bacterial structure (Hamada et al., 2008). Because of the close relationship between dogs and humans, it has been demonstrated that canine *P. gulae* may be transferred to the human oral cavity through close contact (Yamasaki et al., 2012; Bai et al., 2023) and may be hazardous (Inaba et al., 2019, 2020; Urmi et al., 2021; Shimizu et al., 2022). This proves that the prevention and control of periodontal disease and the suppression of the spread of the pathogen are of public health and safety significance. However, there are also differences in the oral microbiota of humans and dogs (Dewhirst et al., 2012; Cunha et al., 2018), so we need to study the pathogenic microbiota of canine PD in more depth. Dental plaque microbes play a crucial role in PD development (Abdulkareem et al., 2023). In the Chinese research landscape, dominant bacterial genera in canines with PD are not extensively documented, and geographical variations in bacterial microbiota associated with PD suggest the need for a deeper understanding within the Chinese context (Nath et al., 2022).

The potential risk of transferring antibiotic-resistant bacteria from dogs to humans underscores the need for careful consideration in selecting therapeutic agents for canine PD (Šakarnytė et al., 2023). This aligns with the global issue of antibiotic resistance, emphasizing the importance of finding alternative treatment options. Epigallocatechin gallate (EGCG), a key component of green tea polyphenols’ catechins, exhibits antibacterial, antioxidant, anti-inflammatory, and anti-tumor effects (Asahi et al., 2014; Chakrawarti et al., 2016). Various catechin components of tea polyphenols have demonstrated efficacy in human PD treatment (Sitheeque et al., 2009; Cui et al., 2012). Given analogous clinical manifestations of PD in canines and humans, the hypothesis that EGCG could serve as a viable alternative to antibiotics is reasonable, addressing the potential risk of antibiotic resistance transfer and exploring a natural compound for managing PD in canines.

Building upon our laboratory’s established research foundation (Bai et al., 2023), our current study aims to collect canine gingival crevicular fluid (GCF) samples for comprehensive microbiota characterization in Beijing using high-throughput 16S rRNA gene sequencing. Subsequent steps involve isolating dominant bacteria from the oral cavities of canines affected by PD and exploring EGCG’s inhibitory potential on these bacteria through *in vitro* antimicrobial sensitivity tests. We plan to construct a PD mice model to evaluate the *in vivo* therapeutic effects of EGCG, assessing alveolar bone loss (ABL), alterations in inflammatory factor expression, and shifts in bacterial microbiota. Our study aimed to assess whether EGCG could serve as a preliminary treatment to potentially become an effective drug in treating canine PD, upon identification of the primary pathogens of canine PD in China.

## 2 Materials and methods

### 2.1 Sample collection

Gingival crevicular fluid (GCF) samples were procured using dental absorbent paper points (0.04 taper, GAPADENT, China) from a cohort of 96 canines, with or without PD, attending the China Agricultural University Veterinary Teaching Hospital from December 2020 to February 2022. Among these, 24 samples (ranging from 8 months to 15 years of age) were utilized for the acquisition of bacterial genera exhibiting high abundance in canine GCF through high-throughput 16S rRNA gene sequencing. The remaining 72 samples were dedicated to bacterial culture. Importantly, none of the canines had undergone antibiotic treatment or exhibited systemic chronic diseases in the 3 months preceding the sampling period.

### 2.2 High-throughput sequencing

GCF samples were gathered, and DNA extraction was performed utilizing a nucleic acid extraction kit (Fast DNA Stool KitM, The Beijing Genomics Institute, China). The resulting DNA samples underwent a nucleic acid concentration assay using the Equalbit 1 × dsDNA HS Assay Kit (Vazyme, Nanjing, China), and the nucleic acid concentration and quality were determined using a Qubit fluorometer (Thermo Fisher Scientific, United States) with the dsDNA high sensitivity assay program. Samples exhibiting superior DNA concentration and quality were prioritized for subsequent Illumina MiSeq sequencing. The ensuing data underwent both statistical and biological analyses using Quantitative Insights into Microbial Ecology (QIIME, V 1.9.1), yielding operational taxonomic unit (OTU) analysis results.

### 2.3 Strains and culture conditions

The collected GCF samples were vortex shaken and isolated using disposable loops (Biologix, Shandong, China) on 10% brain-heart infusion (BHI) sheep blood plates (Solarbio, Beijing, China) containing 5 μg/mL hemoglobin chloride, 1 μg/mL vitamin K1 (VK1), 0.4 mg/mL L-cysteine hydrochloride and 5 mg/mL yeast extract (Solarbio, Beijing, China). The incubation took place at 37°C with 80% humidity under anaerobic conditions (10% CO2, 10% H2, and 80% N2) within an anaerobic culture vessel (YY-s Plus, LongFuJia Biotechnology, Beijing, China) for a culture period of 7–15 days. Single colonies were meticulously chosen and subjected to purification once more. The resulting single colonies were inoculated with a 5% fetal bovine serum (FBS) BHI liquid medium containing hemoglobin chloride and VK1 to be enriched and stored at −80°C. American Type Culture Collection (ATCC) 51700 *P. gulae* was purchased as a quality control bacterium.

### 2.4 16S rRNA sequencing

Polymerase chain reaction (PCR) was performed using KOD-one Mix (25 μL, TOYOBO, Japan) with bacterial universal primers 27F and 1492R. Following agarose gel electrophoresis, the band size and uniqueness of the PCR products were determined, and the PCR products were sent to GENEWIZ^1^ for sequencing based on the Sanger sequencing method. The obtained sequences were compared with those available from the GenBank database using the Ape program on The Basic Local Alignment Search Tool (BLAST).^2^ The results of the comparison with known sequences of strains with >99% similarity were considered as the same bacteria, and the results were accurate to the species level.

### 2.5 Antimicrobial susceptibility testing *In vitro*

Referring to the 9th edition of the standard Methods for Antimicrobial Susceptibility Test of Anaerobic Bacteria published by the Clinical & Laboratory Standards Institute (CLSI) in 2018, drug susceptibility tests were performed according to the procedures of the test for anaerobic bacteria, and the minimum inhibitory concentration (MIC) for EGCG was determined for the clinically dominant isolate and ATCC 51700 standard bacteria in canine GCF samples. EGCG with purity (HPLC) ≥98% were purchased from Sigma (USA). A stock solution of 12.5 mg/mL was prepared by adding 50 mg of EGCG powder to 4 mL of ultrapure water. The drug concentrations in the EGCG-containing drug-sensitive plates were 1.250 mg/mL, 0.625 mg/mL, 0.312 mg/mL, 0.156 mg/mL, and 0.078 mg/mL from highest to lowest.

### 2.6 PD model construction

The research was conducted by internationally accepted principles for laboratory animal use and care. All animal studies were reviewed and approved by the China Agricultural University Laboratory Animal Welfare and Animal Experimental Ethical Committee (Approval ID: AW31103202-2-4). Thirty-three male C57BL/6 mice (Beijing Vital River Laboratory Animal Technology Co., Ltd.), 7–8 weeks old were prepared. The mice were anesthetized by intraperitoneal injection of 10% chloral hydrate (Wabcan, Fujian, China), and the PD model was established by ligating the maxillary second molars using the silk ligature method (Rafael et al., 2018; Chipashvili and Bor, 2022). Group A: saline negative control group, group B: chlorhexidine (Maidihai, Beijing, China) positive control group, group C: low-dose EGCG (0.312 mg/mL) dosing group, group D: medium-dose EGCG (0.625 mg/mL) dosing group, and group E: high-dose EGCG (1.250 mg/mL) dosing group. On the eighth day after modeling, sterile cotton swabs soaked with the drug were inserted into the mouths of the mice and held for 2 min for oral mucosal administration. After 7 days of continuous administration mice were sacrificed and whole blood and bilateral maxillae were collected for subsequent testing.

### 2.7 Evaluation of ABL

Unilateral mouse maxillary bone specimens were soaked and rinsed in hydrogen peroxide and saline (Solarbio, Beijing, China), and the soft tissues attached to the bone tissue were removed as much as possible under a dissecting microscope (Olympus GX71, Japan). The specimens were stained with 0.1% methylene blue (Solarbio, Beijing, China) for 45 s and dried. Unilaterally isolated mouse maxillary specimens were photographed under a dissecting microscope. The distance from the cementum-enamel junction to the alveolar bone crest (CEJ-ABC) was measured using ImageJ software (V 1.47), and measurements were repeated three times at each site.

### 2.8 Microbial evolution in the oral cavity of PD mice

Two filaments filled with GCF for modeling were carefully cut from both sides of the maxillary second molars of mice and placed in 1 mL of BHI liquid medium and vortex shocked. The quantitative liquid was aspirated for anaerobic incubation and colony counting, and individual clones with similar colony morphology and staining characteristics were selected for strain identification.

### 2.9 Morphometric of micro-CT analysis and dental X-ray examination

X-ray micro-computed tomography (Skyscan 1,276, Bruker, Belgium) was used. The mouse maxilla was scanned at a voltage of 60 kV and a current of 200 μA with a scan resolution of 6.5 μm. As the filament was placed on the second molar, the region of interest (ROI) for 3D reconstruction was defined as the area above the root tip below the crown of the second molar, with a volume of 0.5 mm × 0.25 mm × 0.4 mm. Three-dimensional image reconstruction was performed using N-Recon software (V 1.7.4.6). Three-dimensional analysis was performed using CT-AN software (V 1.19.11.1+).

### 2.10 Enzyme-linked immunosorbent assay

Mouse whole blood samples were stored at 4°C overnight, centrifuged at 1000 x g for 20 min, and the supernatant removed to obtain serum samples. The supernatants were then collected and the concentrations of interleukin (IL)-6 and tumor necrosis factor (TNF)-α were determined using enzyme-linked immunosorbent assay (ELISA) kits (Mlbio, Shanghai, China) according to the manufacturer’s protocols. The 96-well plate was read at 450 nm on an enzyme marker for result interpretation (Thermo Scientific, Multiskan Go, United States). All assays were performed in triplicate.

### 2.11 Histological analysis for alveolar bone

The unilateral mouse maxilla was dissected free of soft tissues, immersed in 4% paraformaldehyde fixative (Solarbio, Beijing, China), and fixed at room temperature for 48 h. After decalcification with 10% ethylene diamine tetraacetic acid (EDTA) (Solarbio, Beijing, China) at pH 7.4 for 14 days, the tissues were dehydrated, transparentized, embedded, sectioned, dewaxed, stained with hematoxylin–eosin (HE) and sealed with neutral gum. The samples were ready for microscopic examination and photography.

### 2.12 Statistical analysis

Data were analyzed and measurements were expressed as mean ± standard deviation (SD). Significant differences were determined by *T*-test between two groups and one-way analysis of variance (ANOVA) for more than two groups in Prism (GraphPad Software, V 9.0, United States). *p* < 0.01 was considered significant for differences.

## 3 Results

### 3.1 The bacterial composition of GCF samples from canines based on 16S rRNA high-throughput sequencing

**Figure 1:**
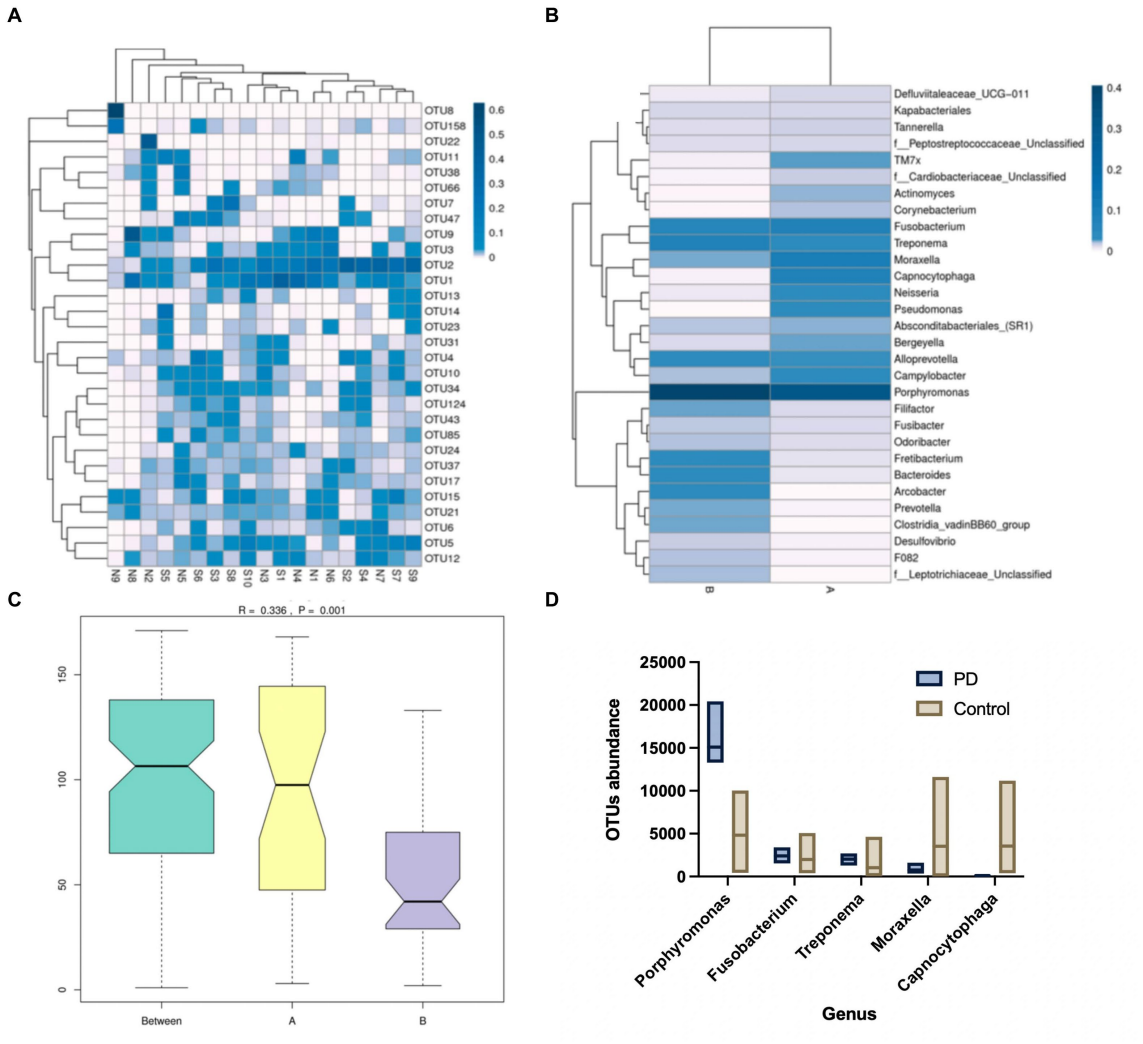
**(A)** Operational taxonomic units (OTU) enrichment clustering heat map. The 30 OTUs with the highest abundance in the sample sequencing results were plotted in R. The corresponding OTU abundance of the 19 samples of gingival crevicular fluid (GCF) were collected. **(B)** Species relative abundance graphs. The samples were grouped accordingly, and the abundance of genera was compared between the two groups. **(C)** Analysis of similarities (Anosim). The size of the between-group and within-group differences were compared between the two sample groups by Anosim analysis. **(D)** Metastatic difference analysis. A comparison of the abundance of the five genera between the two groups of samples has been shown.

GCF samples were collected from 24 canines. A total of 24 samples of GCF were initially tested for this study and 19 samples were ultimately suitable for the machine based on quality control. It was found that among the 19 samples, operational taxonomic unit (OTU) 1 (*Fusobacterium*), OTU2 (*Porphyromonas*), and OTU15 (*Treponema*) exhibited higher abundances, with OTU2 (*Porphyromonas*) being the darkest in the plot and showing the highest relative abundance. The relative abundance of *Moraxella* spp.*, Clostridium phagocytophilum, Neisseria* spp.*, and Pseudomonas* spp. in the GCF of group A (healthy canines) was observed to be higher and the relative abundance of *Porphyromonas* spp.*, Dictyostelium* spp.*, Fretibacterium* spp.*, Mycobacterium* spp., and *Archaea* spp. in the GCF of group B (PD canines) was higher (Figure 1).

### 3.2 Clinically isolated strains of PD canines

**Figure 2:**
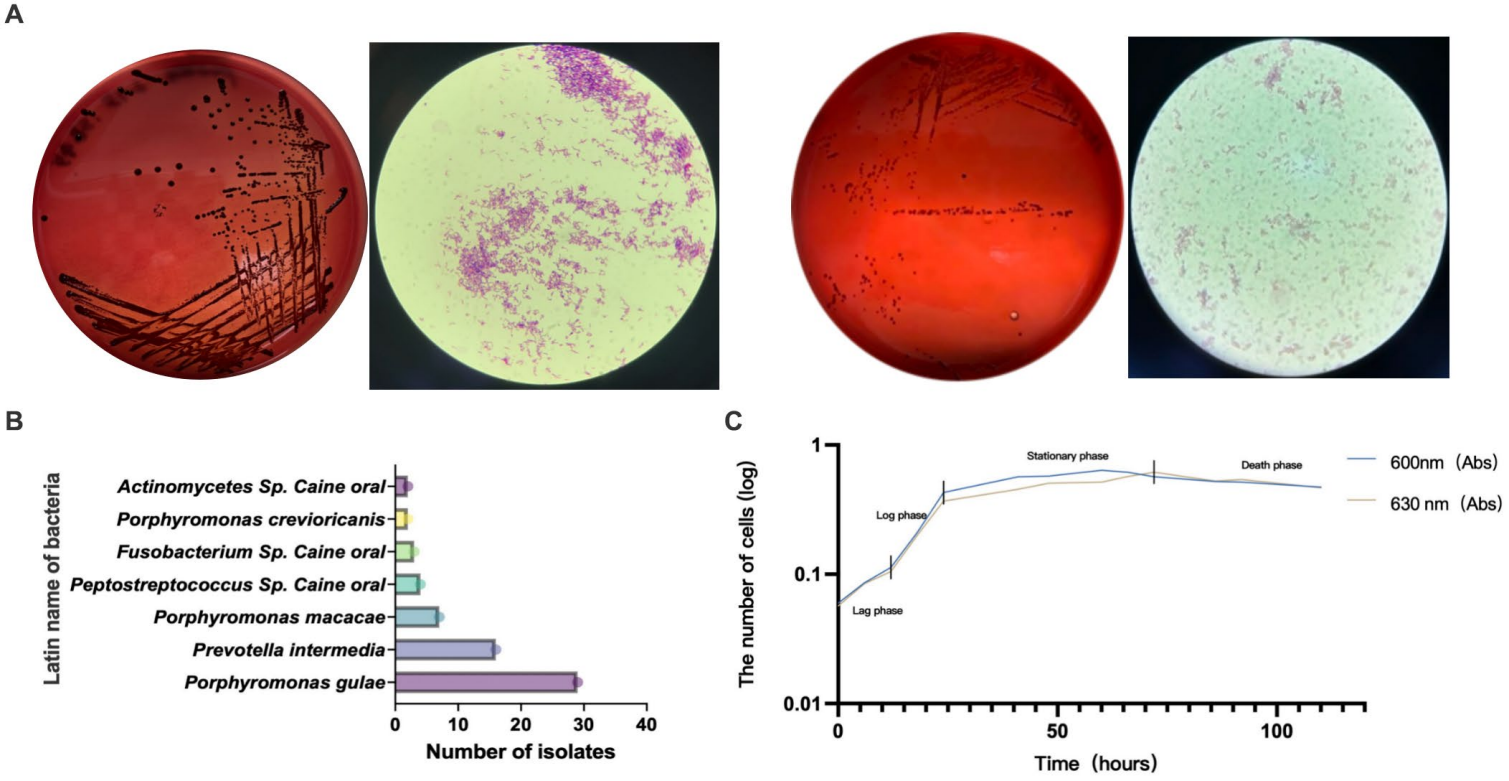
**(A)** Isolated and purified melanin-producing bacteria from the oral cavity of canines with periodontal disease (PD). The modified brain-heart infusion (BHI) plates were incubated anaerobically, then cells were Gram-stained and examined by microscopy (100 X magnification). **(B)** Abundance of various bacteria detected in clinical isolates from the GCF of canines with PD. **(C)** The growth curve of *Porphyromonas gulae* (*P. gulae*) standard bacteria ATCC 51700.

GCF samples from 72 canines with PD were collected for anaerobic culture between December 2020 and February 2022, and a total of 22 species and 92 strains of anaerobic bacteria were isolated and purified. Included *P. gulae, Prevotella intermedia, Porphyromonas crevioricanis, Porphyromonas macacae,* and other bacteria from the oral cavity of canines (Figures 2A,B). As the most academically suspected causative agent of canine PD, the detection rate of *P. gulae* was 31.52% (29/92) (Table 1). A total of 110 h of absorbance values were measured of ATCC 51700 *P. gulae* in modified BHI liquid medium, entering the logarithmic growth phase after 12 h, the stationary phase after 24 h, and the death phase after 72 h (Figure 2C). The bacterium entered the logarithmic phase (12 h) with an optical density (OD) of 0.41 and the end of the logarithmic phase (24 h) with an OD of 0.11.

**Table 1:** Isolation and purification of melanin-producing bacteria from the oral cavity of canines with PD.

Species	Number (*N* = 92)	Detection rates (%)
*Porphyromonas gulae*	29	31.52
*Prevotella intermedia*	16	17.39
*Porphyromonas macacae*	7	0.07
*Peptostreptococcus* spp. Caine oral	4	0.04
*Fusobacterium* spp. Caine oral	3	0.03
*Porphyromonas crevioricanis*	2	0.02
*Actinomycetes* spp. Caine oral	2	0.02

### 3.3 Inhibitory effect of EGCG on the growth of clinical isolate strains

**Figure 3:**
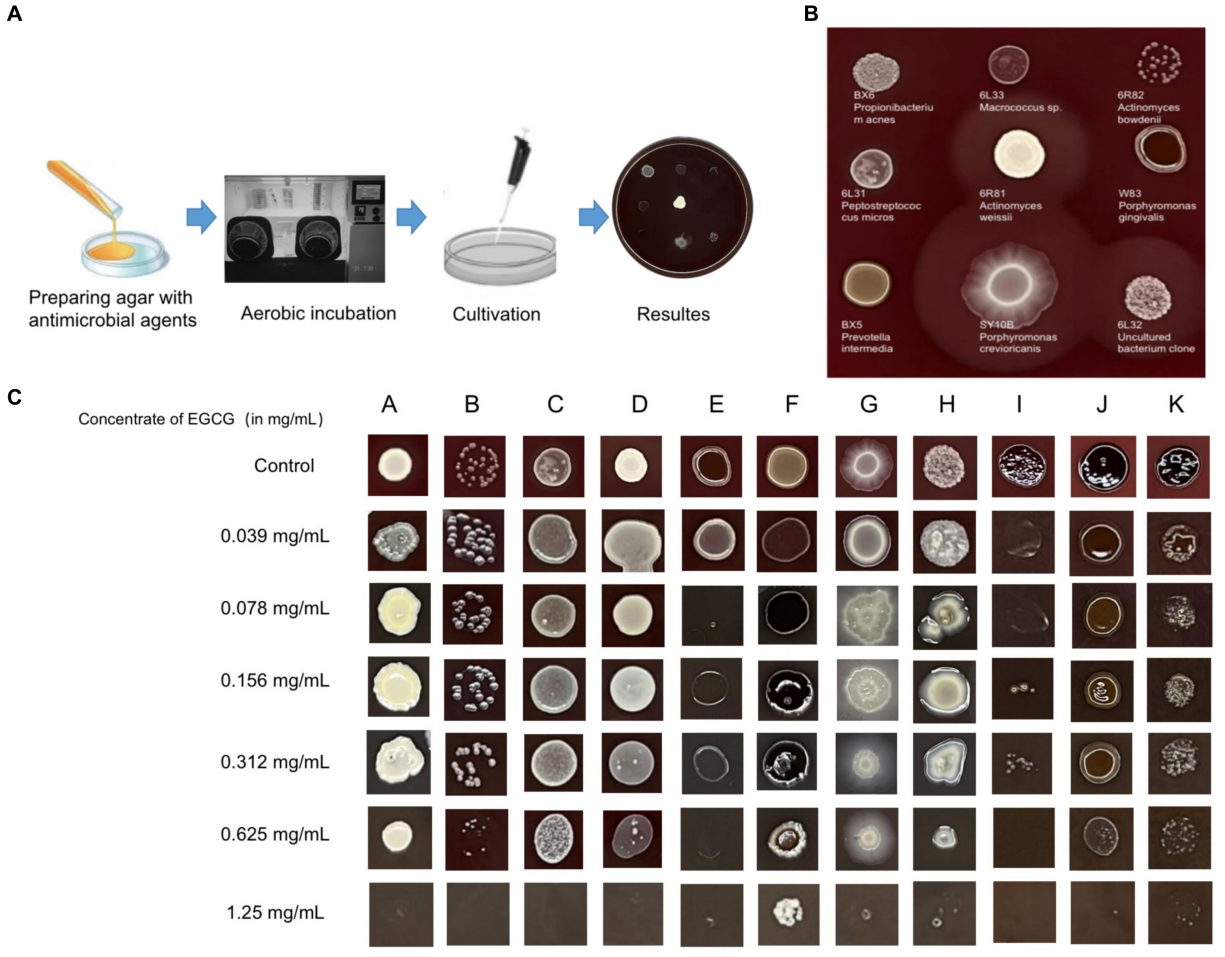
**(A)** Drug sensitivity test used agar dilution method (Wadsworth method) *in vitro*. The drug-containing agar plates were prepared after anaerobic pretreatment. **(B)** For the drug-free control plates, both clinical isolates and standard strains were able to grow well on the enriched Brucella medium. **(C)** Results of the *in vitro* inhibition test for anaerobic bacteria used the Wadsworth method. The lowest drug concentration at which the bacterium was strongly reduced was the minimum inhibitory concentration (MIC) for the bacterium. The graph showed the *in vitro* drug susceptibility results of 11 canine PD-associated strains to EGCG. A: BX6 *Propionibacterium acnes*, B: 6R82 *Actinomyces bowdenii*, C: 6 L31 *Peptostreptococcus micros*, D: 6R81 *Actinomyces weissii*, E: W83 *Porphyromonas gingivalis*, F: BX5 *Prevotella intermedia*, G: SY10B *Porphyromonas crevioricanis*, H: Y1 Uncultured bacterium clone, I: ATCC 51700 *Porphyromonas gulae*, J: SB41 *Prevotella intermedia*, K: SB40 *Porphyromonas gulae*.

An inhibition test using the Wadsworth method *in vitro* on clinical anaerobic isolates from PD canines showed that EGCG was effective against the major human and canine periodontal pathogen, *Porphyromonas gingivalis W83*, and *P. gulae* ATCC 51700 with MICs of 0.078 mg/mL and 0.019 mg/mL (Figure 3). EGCG was also effective against other canine PD isolates. However, 0.05% chlorhexidine, commonly used in veterinary clinical scaling and extraction procedures, showed poor inhibition against some clinical isolates (*SY10B Porphyromonas crevioricanis, 6 L32 uncultured bacterial clone*) (Table 2).

**Table 2:** Inhibition of 17 clinical isolates of canine periodontal disease and 2 standard strains of bacteria by 6 drugs *in vitro*.

Clinical strains of PD canines	EGCG (mg/mL)	CD (mg/mL)	MDZ (μg/mL)	CC (μg/mL)	MER (μg/mL)	AC (μg/mL)
BX6 *Propionibacterium acnes*	1.25	12.5	R	R	0.8	1.28
6 L33 *Macrococcus* spp.	1.25	6.25	R	12.8	0.8	0.64
6R82 *Actinomyces bowdenii*	0.5	3.125	R	3.2	0.8	0.16
6 L31 *Peptostreptococcus micros*	1.25	6.25	R	3.2	0.8	0.16
6R81 *Actinomyces weissii*	1.25	50	R	3.2	0.8	R
BX5 *Prevotella intermedia*	1	12.5	1.6	3.2	0.8	2.56
SY10B *Porphyromonas crevioricanis*	1.25	R	R	3.2	0.8	R
6 L32 Uncultured bacterium clone	1.25	R	R	R	0.8	0.16
W83 *Porphyromonas gingivalis*	0.078	1.56	1.6	6.4	0.8	0.16
Y1 *Fusobacterium* spp. canine oral taxon	1.25	1.56	R	25.6	1.6	R
SY10B *Porphyromonas crevioricanis*	1.25	1.56	1.6	R	0.8	R
6MN *Propionibacterium acnes*	1.25	12.5	1.6	R	6.4	R
SB45 *Prevotella intermedia*	1.25	3.125	R	25.6	1.6	0.16
SB46 *Prevotella intermedia*	1.25	3.125	R	1.6	0.8	0.64
ATCC 51700 *Porphyromona gulae*	0.019	1.56	1.6	1.6	0.8	0.16
SB40 *Porphyromonas gulae*	0.625	6.25	1.6	1.6	0.8	0.16
SB41 *Prevotella intermedia*	0.0625	12.5	1.6	1.6	0.8	0.16
SB42 *Porphyromonas gulae*	0.019	3.125	1.6	1.6	0.8	0.16
SB43 *Prevotella intermedia*	1.25	12.5	1.6	1.6	0.8	0.16

Chlorhexidine, CD; Metronidazole, MDZ; Clindamycin, CC; Meropenem, MER; Amoxicillin and Clavulanate Potassium, AC.

### 3.4 Inhibitory effects of EGCG on the alveolar bone resorption and destruction *in vivo*

**Figure 4:**
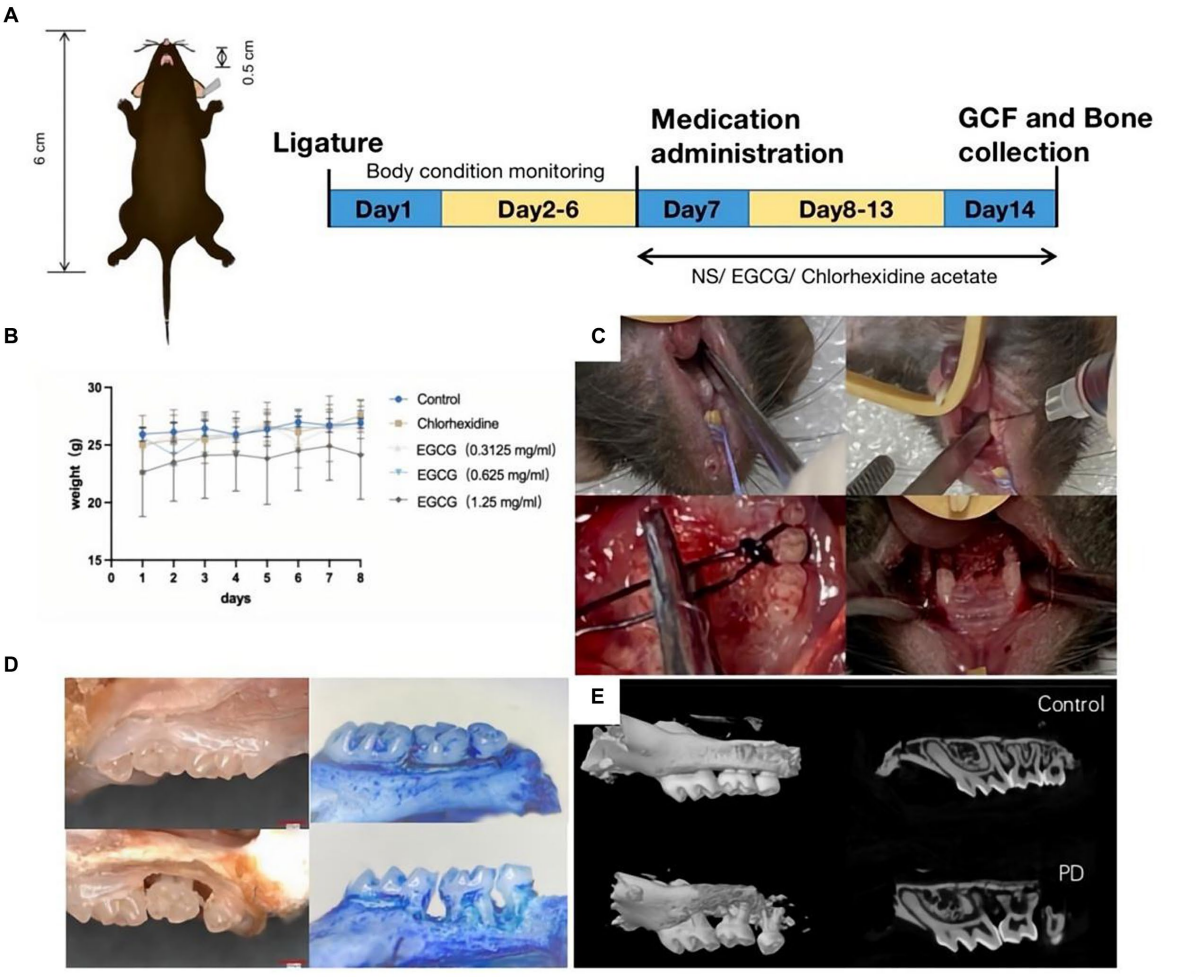
**(A)** Procedure for constructing a PD model in C57BL/6 mice. **(B)** The curve of body weight change curve of the mice after modeling and drug administration. **(C)** Steps of PD modeling by wire ligation. **(D)** Observation of isolated alveolar bone specimens (20X) before and after modeling using a stereomicroscope. **(E)** Observation of alveolar bone loss (ABL) and measurement of bone density using micro-CT.

The animal protocol to assess the therapeutic effect of EGCG on PD *in vivo* involved model establishment on the first day, body weight testing from the second to sixth days, administration initiation on the seventh day, and mouse euthanasia on the fourteenth day. Took GCF samples and maxillary alveolar bone samples from the mice for subsequent tests (Figure 4A). There was no significant change in the body weight of mice before and after administration (Figure 4B). The procedure in the PD mouse model included exposure to the surgical field, expansion of the periodontal space, wire ligation around the maxillary second molar, and repetition of the procedure on the opposite side (Figure 4C). The first row was the isolated tissue of normal mouse maxillary molars, and the second row was the three-dimensional tissue of mouse maxillary molars used to construct a PD model.

The bone volume (BV) in the selected area of the control mice was 0.03333 mm^3^ and the bone mineral density (BMD) was 0.58952 g/cm^3^. The bone volume (BV) in the selected area of the PD mice was 0.00961 mm^3^ and the BMD was 0.18359 g/cm^3^ (Figures 4D,E).

**Figure 5:**
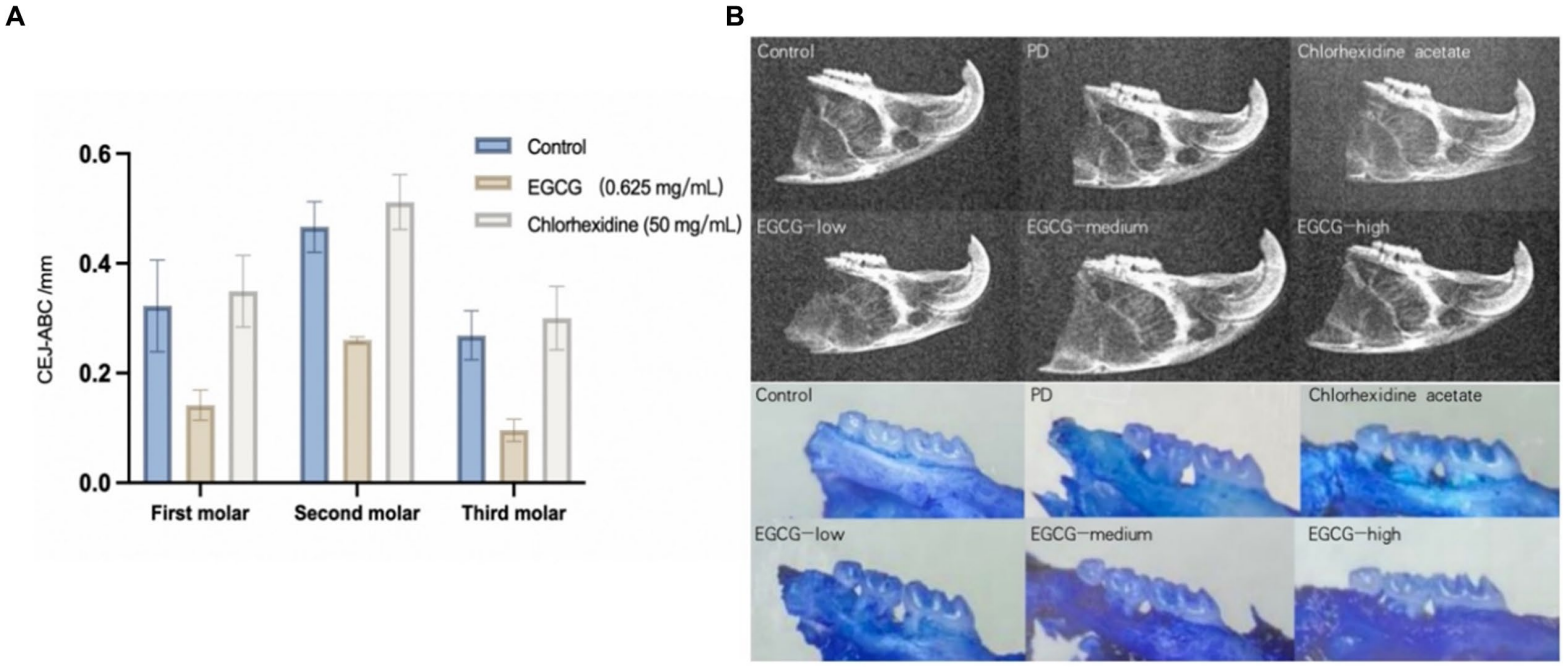
**(A)** Comparison of bone loss distances in PD mice after administration. **(B)** Observation of isolated alveolar bone samples by X-ray versus stereomicroscope.

The mean CEJ-ABC of three molars in the chlorhexidine group was 0.372 mm ± 0.033 mm, the mean CEJ-ABC of three molars in the EGCG group for three concentrations were 0.317 mm ± 0.044 mm, 0.161 mm ± 0.026 mm, and 0. 238 mm ± 0.035 mm in order. The mean CEJ-ABC of the three molars in the saline group was 0.306 mm ± 0.050 mm. There was no significant difference in the CEJ-ABC distance between the saline and chlorhexidine groups (*p*-value = 0.68), and there was also a significant difference in the CEJ-ABC distance between the EGCG and chlorhexidine groups in the mid-dose group (*p*-value = 0.0001). The mid-dose EGCG group was the most effective in improving bone loss and was superior to the chlorhexidine group, an antibacterial rinse commonly used in clinical dentistry (Figure 5A).

**Figure 6:**
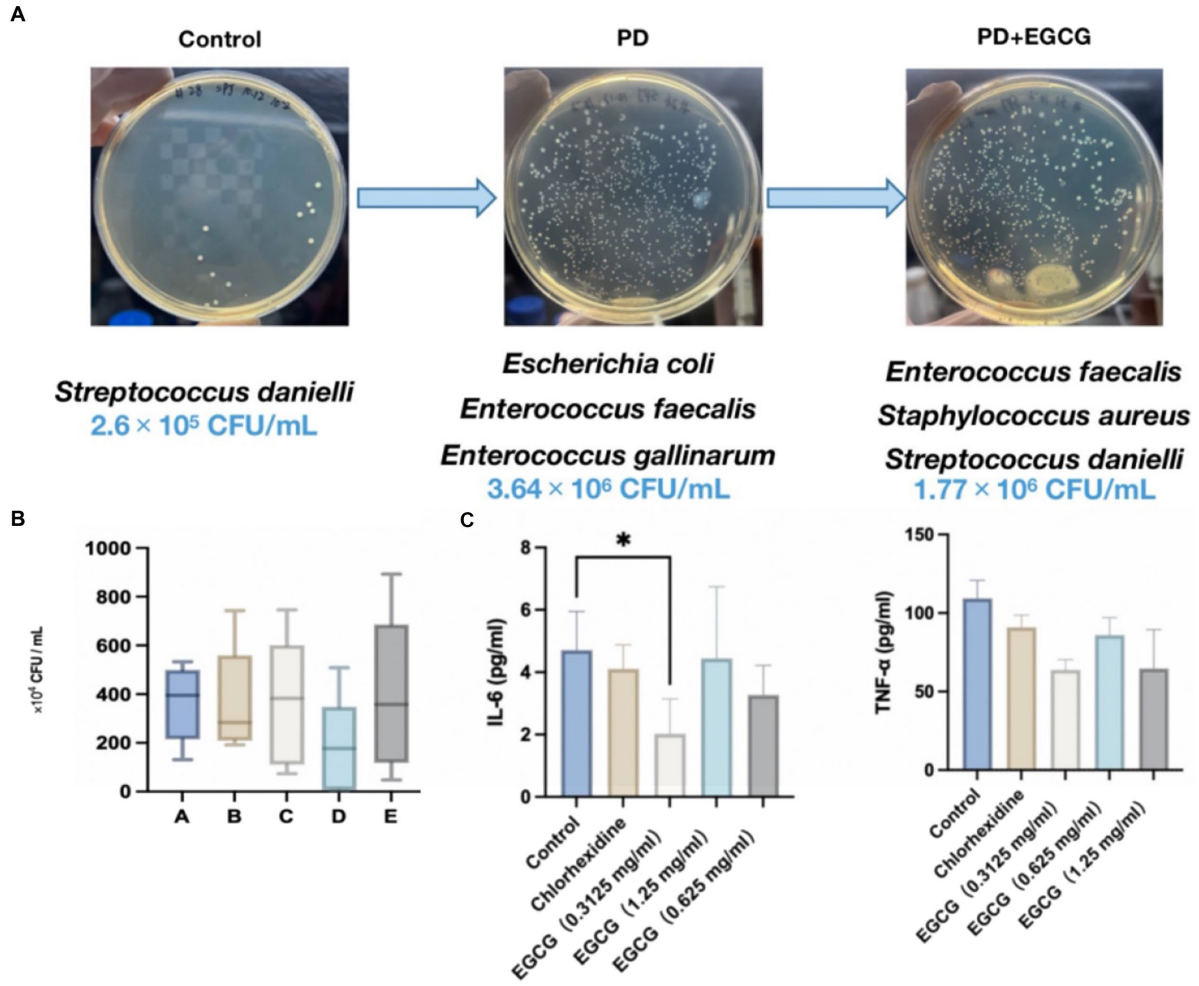
**(A)** Changes in the abundance and species of bacteria in the GCF in the oral cavity of mice before and after modeling. **(B)** Changes in the number of bacteria in the oral cavity of mice after administration. **(C)** Expression levels of the cytokines IL-6, and TNF-α in the serum of mice after administration.

The maxillary alveolar bone of the mice was sectioned, cleaned of periodontal soft tissue, and then stained with 0.1% methylene blue to visualize the ABL. Following the treatment of mice with different periodontal drugs, the effect of the drugs on the ABL was observed using a stereomicroscope. As shown in Figure 6, the morphology of the alveolar bone was found to be intact in the control group, whereas the saline group showed the exposure of the buccal root bifurcation of the second molar and a cup-shaped depression of the resorption surface of the bone centered on the second molar. In the chlorhexidine group, there was severe horizontal ABL in the maxillary molars, with root resorption in the third molar reaching the apical 1/2 area. The effect on ABL varied less between samples for the low, medium, and high EGCG dose groups in the second row (Figure 5B).

### 3.5 Inhibitory effects of EGCG on periodontal inflammatory cytokines and changes in bacterial microbiota *in vivo*

The average number of microorganisms detected in the oral cavity of the mice was 2.6 × 10^5^ CFU/mL, predominantly comprising *Streptococcus dani*, a gram-positive coccus (Figure 6A). In contrast, the average number of microorganisms detected in the oral cavity of the mice without treatment was 3.64 × 10^6^ CFU/mL, mainly *Escherichia coli* and *Enterococcus faecalis*, with a higher proportion of Gram-negative bacteria. After EGCG treatment, the average number of microorganisms detected was 3.61 × 10^6^ CFU/mL, mainly *E. coli, Staphylococcus aureus, Enterococcus faecalis,* and *Streptococcus aureus*, mainly gram-positive coccus. In the high-dose EGCG group, the average number of microorganisms detected was 3.93 × 10^6^ CFU/mL, mainly *E. coli, E. faecalis,* and *Staphylococcus aureus*, mainly gram-positive cocci. These findings indicate that the medium dose of EGCG has a substantial inhibitory effect on oral bacteria in PD mice. Additionally, the microbial community in the oral cavity of mice gradually shifted towards that of healthy mice after EGCG administration (Figure 6A).

Seven days after modeling, mice were grouped for drug treatment. Figure 6B illustrates that both 0.05% chlorhexidine (50 mg/mL) and three different concentrations of EGCG led to a down-regulation of the expression of the inflammatory factors IL-6 and TNF-α in the serum of periodontopathic mice compared to the saline control group. However, only the low-dose EGCG group (0.3125 mg/mL) exhibited a significant difference in the expression of IL-6 compared to the control group (*p*-value < 0.05), with no significant differences observed between the other groups.

### 3.6 Histological analysis for periodontal tissues

**Figure 7:**
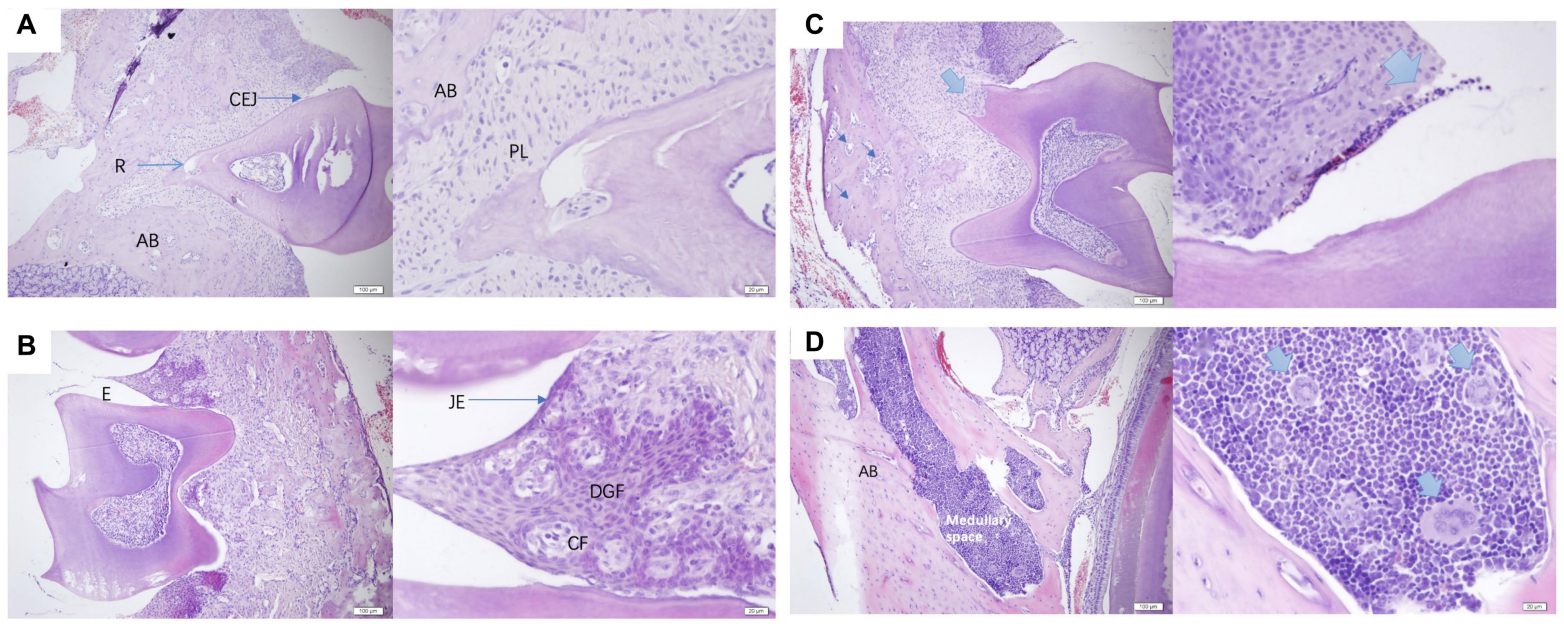
**(A,B)** were normal periodontal sections of mice at a scale of 100 μm and 20 μm. AB, alveolar bone; R, root; CEJ, cementoenamel junction enamel tooth bone boundary; PL, periodontal ligament; E, enamel tooth enamel; JE, junctional epithelium binding epithelium; *CF*, circular fibers; DGF, dentoperiosteal gingival fibers. **(C,D)** were pathological sections of the constructed PD mouse at a scale of 100 μm and 20 μm, alveolar bone, medullary space bone marrow cavity.

The histological analysis of periodontal tissues in healthy mice (Figures 7A,B) revealed intact gingival structures with a slight infiltration of inflammatory cells under the junctional epithelium (JE). This phenomenon is commonly observed clinically around perfectly healthy teeth, representing the body’s defense against the invasion of pathogenic bacteria into the GCF. The gingiva was composed of epithelium and connective tissue, and in the figure, the circumferential fibers (*CF*) and gingival fibers were both composed of collagen type 1. *CF* was present in the connective tissue of the free gingiva and gingival papillae, forming a ring around the cervical part of the tooth. In contrast, PD mice (Figures 7C,D) exhibited osteoblasts and multinucleated giant cells differentiated within the marrow cavity. These cellular components led to a progressive thinning of the bone trabeculae that composed the alveolar bone, accompanied by an increase in the size of the marrow cavity, ultimately resulting in bone loss. The presence of multinucleated giant cells indicated the occurrence of chronic inflammation.

## 4 Discussion

EGCG has been reported to exert anti-inflammatory and antibacterial effects in periodontitis. However, its exact mechanism of action has yet to be determined. Some evidence suggests that EGCG in hPDLFs and hPDLSCs may exert anti-inflammatory effects against *P. gingivalis* lipopolysaccharide, a major pathogen of human periodontitis (Jung et al., 2012). Several studies have demonstrated that EGCG inhibits bone resorption induced by lipopolysaccharides (LPS) by either inhibiting the production of IL-1β or directly impeding osteoclastogenesis (Yun et al., 2004, 2007). Additionally, it has been discovered that EGCG can reduce periapical lesions by inhibiting the expression of cysteine-rich 61 in osteoblasts (Lee et al., 2009). In the field of human dentistry, EGCG has been shown to enhance host conditions in periodontitis and periapical lesions. Such effects stem from EGCG’s bactericidal impact on periodontal pathogens, as well as its inhibitory effect on the cytokine production and inflammatory pathways of gingival fibroblasts or osteoblasts (Okagu et al., 2022; Kováč et al., 2023).

On the suitability of the drug EGCG, it exhibits antioxidant properties by minimizing oxygen radicals and stabilizing compounds (Minnelli et al., 2020). Furthermore, its antitumor effects have been highlighted, elucidating how EGCG inhibits tumor cell growth and induces apoptosis through various cellular pathways, such as regulating the cell cycle, gene expression, signal transduction, and transcription factors (Chen et al., 2019). The role of Nrf2 in regulating detoxifying and antioxidant enzymes, and EGCG’s impact on the Nrf2-ARE signal for inducing antioxidant enzyme expression, leading to an anti-toxic effect, has been emphasized (Na and Surh, 2008). Additionally, EGCG’s antiviral activity, particularly its ability to inhibit the proliferation of the influenza virus in Madin-Darby canine kidney (MDCK) cells, has been underscored (Zhang et al., 2020). However, its relatively underexplored potential in the treatment of canine periodontal disease (PD). Since EGCG has been less studied in this aspect of canine PD, in this study we chose to test EGCG, a natural compound that has not yet been used to treat canine PD, with positive results.

The identified genera that exhibited variations between PD and healthy canines in China include *Porphyromonas*, *Fusobacterium*, *Treponema*, *Moraxella,* and *Capnocytophaga.* A comparative analysis with previous studies underscores noteworthy differences. Notably, at the phylum level, the oral microbiota of healthy canines predominantly comprised *Thickworm* (45.9%), *Anaplasma* (14.7%), *Methanobacterium* (12.2%), *Helicobacter* (10.5%), *Coelenterata* (3.7%), *Actinobacteria* (3.4%), and *Clostridium* (2.8%) (Riggio et al., 2011). Examining the genus level, *Pasteurella, Bergeyella, Porphyromonas*, and *Actinobacter.* exhibited the highest detection rates in healthy canines (Davis et al., 2013). Further, at the species level, dominant species in healthy canines, canines with gingivitis, and those with periodontitis included specific species of *Pseudomonas* (30.9%), *Porphyromonas cangingivalis* (16.1%), and oral desulfurization bacteria (12.0%), respectively (Riggio et al., 2011). Noteworthy species associated with PD encompassed *Moraxella* spp. COT-396, *Bergeyella zoohelcum*, *Neisseria shayeganii*, *Pasteurellaceae* spp. COT-080, *Capnocytophaga* spp. COT-339, and *Stenotrophomonas* spp. COT-224 (Lenzo et al., 2016).

Comparing these findings with past research and incorporating the outcomes of the current high-throughput sequencing study, it is evident that the canine oral cavity harbors a diverse array of microorganisms. Some of these microorganisms remain unexplored and unidentified, underscoring the importance of microbial culture techniques in understanding the etiology of PD in canines, particularly given the historical limitations associated with anaerobic culture methods. This insight further emphasizes the necessity of ongoing studies to elucidate and characterize the oral microbiota in canines comprehensively.

In the subsequent phase of our study, we successfully isolated the clinical strain of *Porphyromonas gulae*. Additionally, the standard bacterium ATCC 51700 *P. gulae* thrived under the established test conditions, affirming the feasibility of its culture. Extensive research findings consistently underscore *Porphyromonas gulae* as the primary causative agent of PD in dogs. Recent studies, including those referenced (Dewhirst et al., 2012), substantiate the pivotal role and pathogenicity of *P. gulae* in canine PD.

The intricate interplay of various bacteria, exemplified by *Porphyromonas* spp., and their byproducts, contributes to the detrimental impact on periodontal tissues. This microbial colonization initiates on the tooth surface and within periodontal pockets, proliferating before progressing to invade the periodontal tissues and potentially entering the bloodstream (Özavci et al., 2019; Urmi et al., 2021; Shimizu et al., 2022). *Porphyromonas* spp. were detected in substantial quantities in the plaque of both healthy and periodontally affected dogs, emphasizing their ubiquitous presence. Notably, the abundance of *Porphyromonas* spp. in the oral cavity of dogs with periodontal issues significantly exceeded that in their healthy counterparts. This aligns seamlessly with the heightened prevalence of *Porphyromonas* spp. observed in both anaerobic culture results and the outcomes of high-throughput sequencing conducted in our study. This consistent correlation reinforces the association between *Porphyromonas* spp., particularly *P. gulae*, and the manifestation of PD in canines.

Furthermore, our investigation demonstrated the inhibitory potential of EGCG against a diverse array of principal pathogens associated with canine PD, notably *Porphyromonas gulae*. EGCG, as a natural active ingredient, boasts a multitude of applications. The discerned antimicrobial and therapeutic efficacy of EGCG surpassed that of the conventional 0.05% chlorhexidine acetate rinse commonly employed in small animal dentistry. This revelation suggests the promising utility of EGCG as a superior alternative for combatting canine PD, offering a potential avenue for enhanced treatment modalities in veterinary oral health.

Moreover, the application of EGCG exhibited significant efficacy within the *in vivo* milieu of our constructed PD mouse model, manifesting notable antibacterial, anti-inflammatory, and bone loss mitigation effects. However, it is imperative to acknowledge two challenges associated with utilizing mice as a method for constructing PD models. Firstly, operational challenges within a confined space posed difficulties, and secondly, the initial high mortality rate of molded mice. Addressing these concerns, strategic modifications such as the reduction of the operation duration, timely rewarming post-operation, and vigilant daily monitoring of body weight contributed to an improved survival rate among the mice. Despite these challenges, the observed therapeutic effects of EGCG, encompassing anti-inflammatory properties, antibacterial activity, and mitigation of bone loss in PD mice, are highly encouraging. These findings provide a foundation for considering the potential therapeutic applications of EGCG in managing PD in canines, extrapolating from the positive outcomes observed in the murine model.

Although there are important discoveries revealed by these studies, there are also limitations. While our study has made noteworthy contributions, we acknowledge certain limitations. The sample size remains constrained, necessitating expansion in subsequent studies for more robust conclusions. Furthermore, our investigation employed a singular mode of drug administration and a specific drug dosage form. To enhance clinical relevance, future studies should explore diverse drug carriers and dosage forms, conducting direct tests on the target animal, the canine. Ongoing modifications, such as nanoparticle preparation and temperature-sensitive hydrogel formulations, are in the pipeline to extend drug retention in the oral cavity of mice, thus improving bioavailability and therapeutic efficacy.

Despite the limitations, we achieved the successful isolation and cultivation of *P. gulae*, the primary causative agent of canine PD. Our laboratory’s accomplishment in isolating a clinical strain of *P. gulae*, a significant breakthrough under strict anaerobic conditions, provides crucial technical support and invaluable strain material for subsequent experiments. Additionally, our study fills a critical gap by elucidating the microbiota differences between PD and healthy canines in China, serving as a foundational resource for future research on the pathogenesis of canine PD and drug screening.

## 5 Conclusion

Our research demonstrates the significant antibacterial effects of Epigallocatechin-3-gallate (EGCG) against the prevalent bacterium *P. gulae* in canine periodontal disease (PD). Moreover, EGCG exhibits anti-inflammatory properties and proves effective in addressing bone loss in a PD mouse model. These findings collectively suggest the therapeutic potential of EGCG in the treatment of canine PD. The outcomes of this study contribute valuable data, laying the foundation for further exploration and screening of alternative antibiotic drugs to advance the management of canine PD.

## Data availability statement

The original contributions presented in the study are included in the article/supplementary materials, further inquiries can be directed to the corresponding authors.

## Ethics statement

The animal studies were approved by China Agricultural University Laboratory Animal Welfare and Animal Experimental Ethical Committee (Approval ID: AW31103202-2-4). The studies were conducted in accordance with the local legislation and institutional requirements. Written informed consent was obtained from the owners for the participation of their animals in this study.

## Author contributions

PS: Writing – original draft, Writing – review & editing. YH: Formal analysis, Writing – review & editing. DL: Project administration, Writing – review & editing. YJ: Funding acquisition, Project administration, Supervision, Writing – review & editing. JL: Conceptualization, Funding acquisition, Supervision, Writing – review & editing.

## Footnotes

^1^ https://www.genewiz.com.cn

^2^ https://blast.ncbi.nlm.nih.gov/Blast.cgi
